# Conduction system pacing: what emerging evidence and what indications?

**DOI:** 10.1093/europace/euag181

**Published:** 2026-07-20

**Authors:** Haran Burri, Anja Zupan Mežnar, Klaus K Witte

**Affiliations:** Cardiac Pacing Unit, Cardiology Department, University Hospital of Geneva, Geneva, Switzerland; Cardiology Department, University Medical Centre Ljubljana, Ljubljana, Slovenia; Leeds Institute for Cardiovascular and Metabolic Medicine, School of Medicine, University of Leeds, Leeds, UK

**Keywords:** Conduction system pacing, His bundle pacing, Left bundle branch area pacing, Biventricular pacing

## Abstract

Conduction system pacing is a major milestone in cardiac stimulation and is being increasingly adopted as a more physiological alternative to right ventricular and biventricular pacing. This expert viewpoint discusses the current evidence, indications and remaining challenges of this promising therapy.

## Graphical Abstract

**Graphical Abstract:**
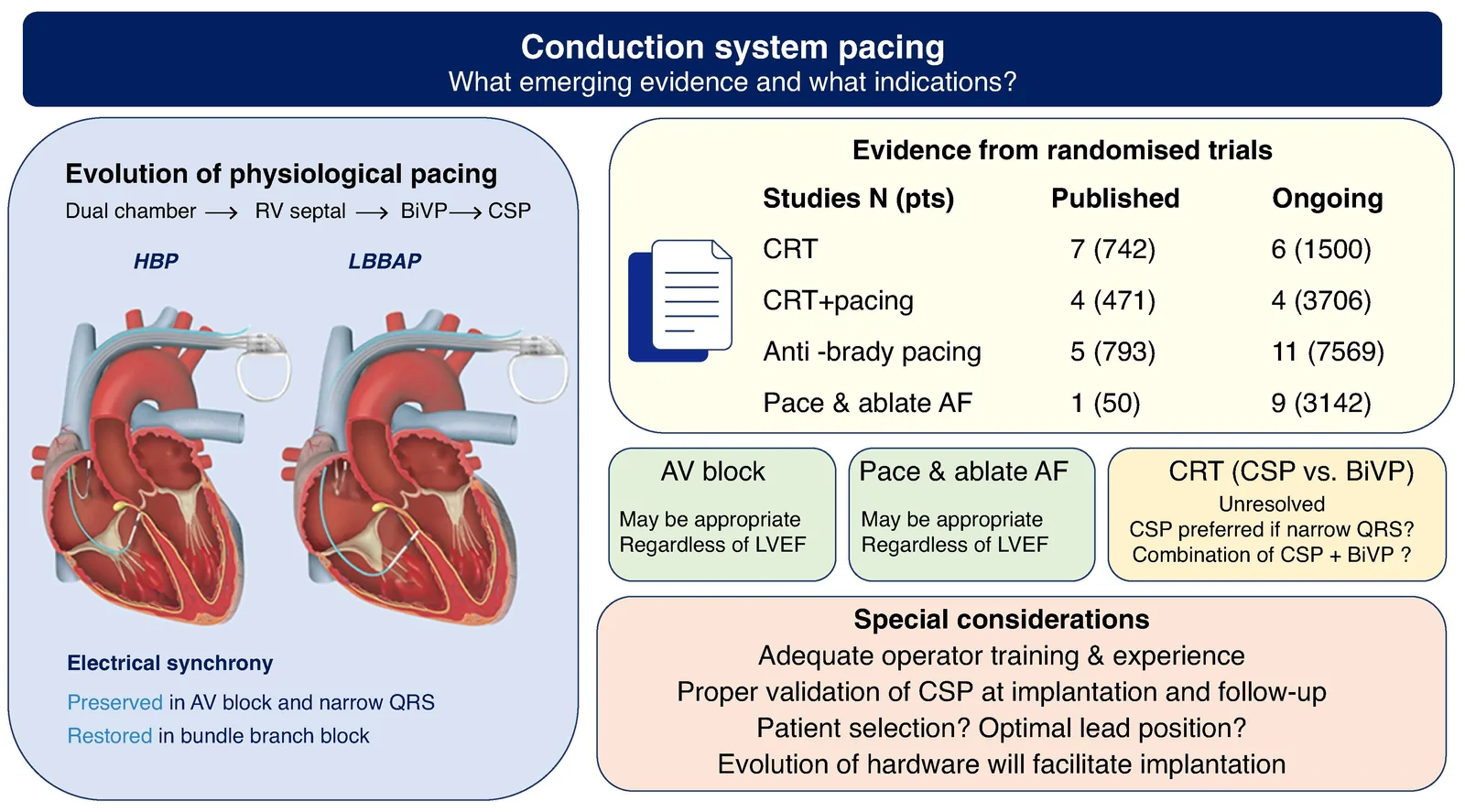
Overview of conduction system pacing with body of evidence from published and number of ongoing randomized trials (the latter including ≥80 patients)—number of trials are indicated, with numbers of patients displayed in brackets. Current indications and principal special considerations are also shown. Abbreviations: AF, atrial fibrillation; AV, atrioventricular; BiVP, biventricular pacing; CRT, cardiac resynchronization therapy; CSP, conduction system pacing; HBP, His bundle pacing; LBBAP, left bundle branch area pacing; LVEF, left ventricular ejection fraction; RV, right ventricular.

The quest for physiological pacing has evolved over the last decades from dual-chamber pacing, to right ventricular septal pacing and to biventricular pacing (BiVP). Cardiac resynchronization therapy (CRT) with BiVP has proven successful in treating selected patients with heart failure, reduced left ventricular ejection fraction (LVEF) and electrical dyssynchrony. However, its benefit in patients with atrioventricular block (AVB) as an alternative to right ventricular pacing (RVP) has been disappointing,^1^ and challenges remain, such as phrenic nerve capture, dependence on coronary sinus venous anatomy, and elevated capture thresholds.

Conduction system pacing (CSP), comprising mainly of His bundle pacing (HBP) and left bundle branch area pacing (LBBAP), has the potential to facilitate more physiological ventricular activation than the previous pacing modalities, with growing evidence of safety and efficacy (see *Figure 1*). By engaging the intrinsic conduction system, it not only *preserves* physiological ventricular electrical activation in patients with AVB and a narrow QRS, but can also *restore* electrical synchrony in patients with bundle branch block by pacing the conduction system distal to the site of block. There is little doubt that CSP is the closest we have come so far to reproducing normal ventricular activation by electrical stimulation.

**Figure 1:**
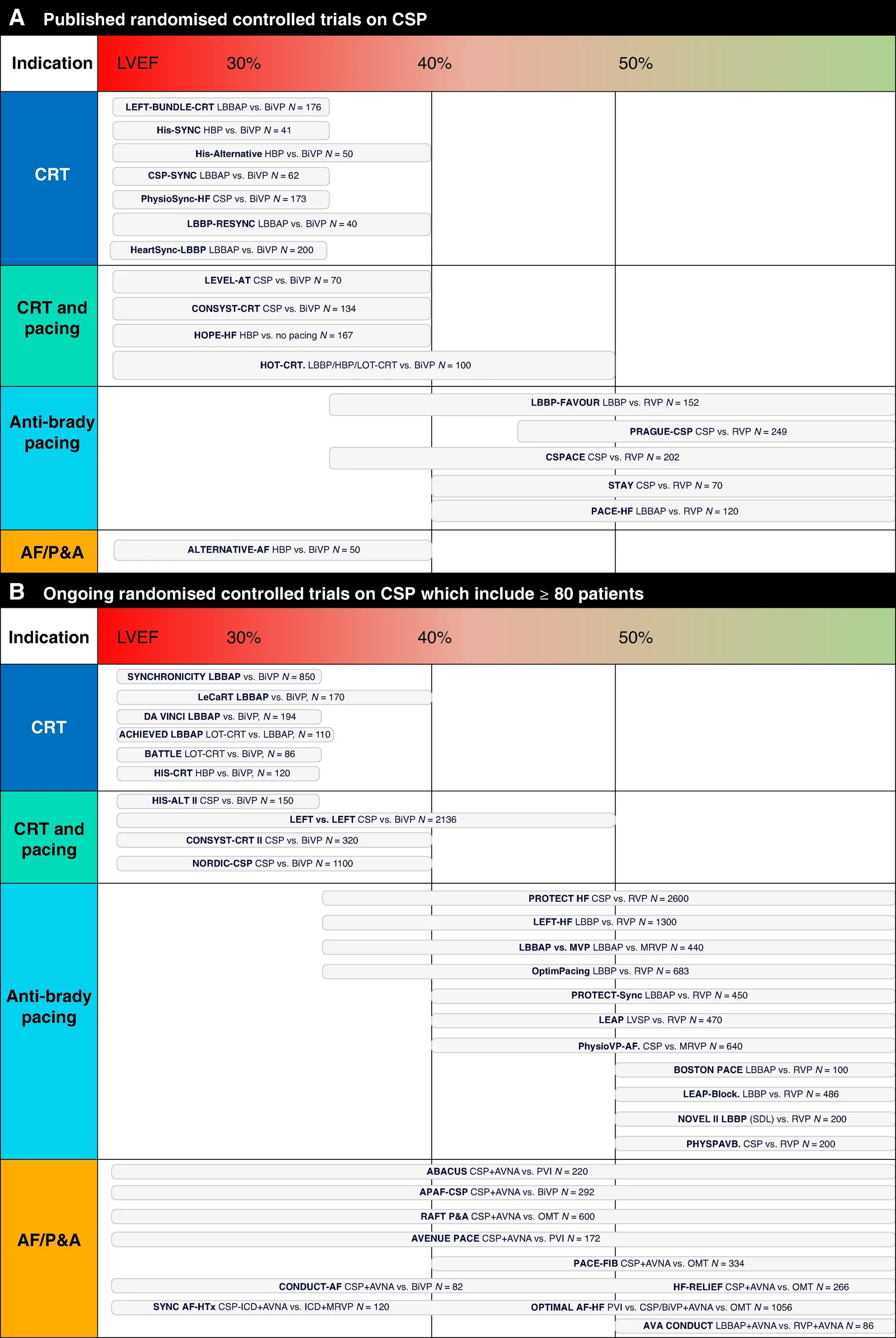
(*A*). Published randomized controlled trials on CSP. (*B*). Ongoing randomized trials on conduction system pacing which include ≥80 patients (according to ClinicalTrials.gov accessed on 28 June 2026). AVNA, atrioventricular nodal ablation; BiVP, biventricular pacing; CSP, conduction system pacing; HBP, His bundle pacing; ICD, implantable cardioverter defibrillator; LBBAP, left bundle branch area pacing; LOT-CRT, left bundle opitmized cardiac resynchronization therapy; LBBP, left bundle branch pacing; LVSP, left ventricular septal pacing; MRVP, minimized right ventricular pacing; OMT, optimal medical therapy; PVI, pulmonary vein isolation; RVP, right ventricular pacing; SDL, stylet-driven leads.

The most physiological pacing modality is HBP, which was introduced a quarter of a century ago. However, it is associated with loss of CSP in about 20% of patients, mainly due to rising thresholds and may be associated with sensing issues.^2^ These may be mitigated by obtaining a His potential current of injury and testing lead stability.^3^ His bundle pacing is still employed as a primary strategy by a minority of CSP implanters, but is most often used as a backup solution when LBBAP yields suboptimal results. The currently favoured form of CSP is LBBAP, due to its favourable electrical parameters and its perceived ease of implantation.

The indications for CSP have been defined in a 2025 European Heart Rhythm Association (EHRA) consensus document.^4^ This document states that CSP may be appropriate for treating patients with AVB, regardless of LVEF. Most of the patients included in the randomized trials evaluating CSP (all with <250 patients) had preserved ejection fraction. A recent registry-based trial included 344 patients <65 years and reported significantly fewer heart failure hospitalizations at 2 years with CSP compared with RVP.^5^ The MELOS-RELOADED study included 3382 propensity-matched patients with LVEF >40% and >20% ventricular pacing, and reported that LBBAP was associated with lower mortality than RVP [hazard ratio 0.53, 95% confidence interval (CI) 0.42–0.65, *P* < 0.001, and an absolute reduction of 11.8% after 4 years of follow-up, *P* < 0.001].^6^ In contrast, the Biopace trial found no benefit of BiVP compared to RVP in 1810 patients with a Class I indication for pacing, irrespective of heart failure status, QRS duration, LVEF (mean 55.4 ± 12.2%), or baseline rhythm.^1^ Relatively few patients with advanced AVB and reduced LVEF have been included in randomized trials, with a total of <300 subjects testing BiVP and none for CSP.^4^ Many physicians favour CSP over BiVP in AVB patients with a narrow QRS, although operator experience should be taken into account.

As with AVB, the EHRA consensus document states that it may be appropriate to implant CSP in patients with a pace and ablate indication, irrespective of LVEF. The Alternative AF study^7^ randomized 50 patients with atrial fibrillation, LVEF ≤40%, and a narrow QRS or right bundle branch block to receive either HBP or BiVP for 9 months in a crossover design, and found that LVEF was significantly higher during HBP. However, in the setting of atrioventricular nodal ablation, LBBAP is generally favoured over HBP due to the proximity of the ablation site to the lead tip (which risks compromising lead function).

The situation is more complex in the setting of CRT. Here, BiVP has proven to be effective in improving patient outcomes in trials spanning two decades, including >10 000 patients. Whether CSP does just as well (or better) is currently unresolved.^8^ A recent retrospective longitudinal cohort of Medicare pacemaker patients and a CRT indication implanted with BiVP (*n* = 5693) or CSP (*n* = 2207), found that the latter therapy was non-inferior in terms of safety and effectiveness, with lower total mortality (despite these patients appearing sicker than those with CRT).^9^ The randomized trials comparing CSP with CRT which are currently available are of relatively small size (≤200 patients) and show equivocal results.^10–12^ The HeartSync-LBBP trial conducted in China randomized 200 patients with left bundle branch block (LBBB) and LVEF ≤35% to either BiVP or LBBAP.^11^ Implant success of LBBP with confirmed conduction system capture was 98% (and 75.5% of patients demonstrated transition in QRS morphology with decrementing output), which is by far the highest ever reported in this population of patients with a challenging substrate. The hazard ratio for mortality or heart failure hospitalization (HFH) was 0.26 (95% CI 0.12–0.57, *P* < 0.001), mainly driven by the latter endpoint. In contrast, the Physio-Sync HF trial,^12^ conducted in Brazil, randomized 173 patients with LBBB and LVEF ≤35%, and found significantly worse composite clinical and echocardiographic outcomes with CSP compared to BiVP, as well as worse mortality and HFH (hazard ratio 2.35, 95% CI 0.99–5.61). It is important to note that in this study, many operators were relatively inexperienced (43% had performed <40 CSP cases), and 18% of the CSP group received deep septal pacing rather than LBBAP. Furthermore, it is unknown how many additional patients experienced loss of conduction system capture due to microdislodgements (reported elsewhere in up to 13.5% of patients^14^), as follow-up ECG data were not reported. The ChiCSP study^13^ indicated that conduction system capture is important for achieving good outcomes with CSP in patients with a CRT indication, and that left ventricular septal pacing is suboptimal. Deep septal pacing, which is somewhat akin to RVP, may even be detrimental in patients with ventricular dysfunction.

The above-mentioned trials underscore the importance of properly performing CSP according to rigorous standards. The updated EHRA curriculum for the heart rhythm specialist now includes CSP.^14^ The EHRA consensus document for CSP implantation provides a framework for performing the technique and the criteria to validate LBBAP.^3^ Unlike HBP, where CSP capture is unambiguous, successful LBBAP implantation may be more prone to misinterpretation, hence the greater perceived ease of implantation. Proper training and adequate equipment to record endocardial signals and the 12-lead ECG are paramount. However, this does not mean that these procedures should be reserved for tertiary or highly specialized centres. Portable equipment is already available which can replace electrophysiology recording systems if the latter are not available. Evolution in lead design and implant tools will facilitate effective delivery of CSP, as was the case with coronary sinus leads. More evidence from randomized clinical trials, which is currently underway (see *Figure 1B*), is necessary to confirm the role of CSP with respect to RVP and BiVP in future guidelines. A caveat of these large trials is that centres with less experience are also included to reach the targeted numbers, thereby possibly compromising the quality of the therapy delivered and replicating the situation encountered with the Physio-Sync trial. It will therefore be essential that these trials carefully evaluate ECGs at implantation and at follow-up by core labs, to adjudicate which therapy was actually delivered. On the other hand, such trials will inform us of the applicability of CSP, given that most centres implanting pacemakers for bradycardia are not specialized pacemaker units.

In the meantime, CSP offers us additional treatment options. This is illustrated by the LEFT-BUNDLE CRT randomized trial, which failed to show non-inferiority of LBBAP compared to BiVP in 177 CRT candidates in the ‘intention to treat’ analysis.^15^ The response rate in the BiVP group was 95% (based on symptoms and echocardiographic remodelling), surpassing that of any other CRT trial reported so far. This was most likely because a suboptimal coronary sinus lead position was not accepted, with 20.7% crossovers from BiVP to LBBAP. Response rate was also high in the LBBAP group at 92%, and differences were non-significant in the ‘on-treatment’ analysis.

In addition to CSP being an alternative to BiVP, the treatment modalities may be combined with His-optimized CRT or left-bundle optimized CRT, and thereby be complementary. This modality also mitigates the negative impact of possible lead microdislodgement with loss of CSP capture.

It is important to position CSP based on scientific evidence, which will grow in the coming years. There is still much to learn, including how best to assess conduction system capture and electrical synchrony, where to position the leads and which patients are best suited to each pacing strategy. There has never been a more exciting time to be involved in pacing therapy!

## Data Availability

There are no original data for this viewpoint article.
